# Trust in Digital Sexual Health Information Among Chinese College Students: Qualitative Study

**DOI:** 10.2196/97644

**Published:** 2026-09-15

**Authors:** Ya Shi, Ying Zhu, Peiyu Sun, Ying Ye, Qingqing Nie, Maiwuludai Hasimu, Qingyao Wang, Can Gu

**Affiliations:** 1 School of Nursing Shandong Xiehe University Jinan, Shandong China; 2 School of Medicine Xinjiang University of Science and Technology Korla, Xinjiang China; 3 School of Nursing, Faculty of Medicine Yangzhou University Yangzhou, Jiangsu China; 4 Xiangya School of Nursing Central South University Changsha, Hunan China

**Keywords:** Chinese college students, functional trust, knowledge-action gap, online information seeking, sexual health information

## Abstract

**Background:**

Digital platforms are primary sources of sexual health information for young adults, yet they are widely perceived as unreliable. This tension between distrust and reliance remains undertheorized.

**Objective:**

This study aimed to examine how Chinese college students use online sources for sexual health information under conditions of uncertainty and to explore how they navigate the tension between distrust and reliance.

**Methods:**

We conducted semistructured in-depth interviews with 22 Chinese undergraduate students (aged 18-25 years) recruited from 8 universities across China using a purposive sampling strategy with maximum variation, complemented by snowball sampling. Interviews were conducted in January 2026. Telephone interviews explored participants’ experiences with online sexual health information seeking, including their perceptions of reliability, encounters with contradictory content, credibility evaluation strategies, management of uncertainty, the tension between distrust and continued reliance, and the role of cultural factors in shaping information-seeking behaviors. Data were analyzed using reflexive thematic analysis.

**Results:**

Participants reported frequent reliance on online sexual health information despite widespread concerns about its reliability. Four interconnected themes were identified: (1) a fragmented and unreliable information landscape characterized by abundant but superficial, contradictory, and difficult-to-apply content; (2) incomplete trust yet continued reliance, driven by precautionary risk logic and limited offline alternatives; (3) strategies for constructing confidence through cross-platform verification, experiential validation, and platform-specific evaluation; and (4) hidden costs of navigation, including confusion, information overload, and search abandonment.

**Conclusions:**

Based on these findings, we propose functional trust as an interpretive conceptualization describing how users act on information with sufficient confidence despite persistent uncertainty—as an interpretive lens for understanding how users navigate digital health information. Our findings suggest that the use of online sexual health information does not depend on trust in the conventional sense, but may involve the construction of functional trust under conditions of persistent uncertainty. Addressing challenges in digital sexual health communication requires not only improving information quality but also enhancing its usability and reducing the cognitive burden of navigation within structurally constrained environments.

## Introduction

The digital transformation of health information seeking has fundamentally altered how individuals, particularly young adults, access knowledge about sexual health. Online sexual health information refers to any digital content accessed via the internet (including search engines, social media platforms, AI chatbots, video-sharing sites, and health forums) that addresses topics related to sexual health. This encompasses, but is not limited to, information about contraception, sexually transmitted infections (including HIV/AIDS), reproductive health, pregnancy, sexual anatomy and physiology, sexual orientation and gender identity, sexual pleasure and function, relationship and consent, and prevention of sexual violence. The scope excludes formal telemedicine consultations with verified health care providers, as those involve direct professional interaction, and focuses instead on publicly available, user-generated or commercially produced content that users encounter through self-directed searching.

In China, as elsewhere, a growing body of evidence suggests that the internet has become a primary, and for many the most accessible, source of sexual health information for college-aged populations. A nationwide study of over 20,000 college students found that 92.37% accessed sexual health information through mass communication channels [1]. A separate survey of 12,632 students in Guangzhou reported that 76.96% accessed HIV/AIDS knowledge from social software [2]. More broadly, a national survey of 52,256 youth revealed that media, spanning online platforms and notably pornography, serves as the primary source of sexual knowledge, particularly for male students [3]. These findings, drawn from distinct samples and methodologies, collectively indicate a strong and consistent pattern of digital reliance.

This digital abundance appears, on its surface, to represent a democratization of sexual health knowledge, offering anonymity, convenience, and freedom from the judgment that often accompanies face-to-face inquiry [4-6]. Yet beneath this surface lies a profound paradox: the same digital environment that provides unprecedented access to sexual health information is also characterized by pervasive unreliability. Users encounter contradictory advice, commercial content disguised as education, sensationalized misinformation, and algorithmic amplification of extreme or misleading perspectives [7-9]. We define the “untrusted information environment” as one characterized by uncertainty (contradictory, incomplete, or unverifiable information) and distrust (users’ perception that information is unreliable, even as they continue to use it). In the context of sexual health, where misinformation can lead to harmful health decisions, erode trust in medical institutions, and even enable misinformed policy restrictions [10], this unreliability is not merely an inconvenience but a significant public health concern, as recognized by the World Health Organization and documented across multiple national contexts [11,12].

For young adults navigating the transition to sexual maturity, this paradox generates a fundamental dilemma. They require accurate information to make informed decisions about contraception, infection prevention, and reproductive health, yet the digital environment they predominantly rely on is widely recognized as unreliable. This tension, between the necessity of information and the untrustworthiness of its primary source, creates what we term the “trust paradox”: the simultaneous experience of distrust in online sexual health information and continued reliance upon it.

Existing research has documented the prevalence of digital health information seeking among young adults [13], the challenges of evaluating online health information [14], and the role of social media in shaping sexual health knowledge [15]. A substantial body of literature has also examined trust in health information, focusing on trust in health care providers [16], institutional sources [17], and online platforms [18]. These studies typically conceptualize trust as a prerequisite for information adoption, operating on a linear model in which trust precedes use. However, this linear framework fails to capture the complexity of how individuals engage with digital health information in practice. Emerging evidence suggests that health information seeking in digital environments involves more nuanced dynamics: individuals may use information they do not fully trust when alternatives are scarce [19,20], may selectively trust certain aspects while doubting others [21,22], or may adopt a pragmatic strategy of “satisficing,” settling for information that is sufficient given time and resource constraints [23]. Yet these dynamics remain underexplored in the specific context where the stakes are high, alternatives are limited, and the tension between distrust and reliance is most acute.

The Chinese context amplifies this tension. Despite significant economic development and increasing social liberalization, open discussion of sexuality remains constrained in many families and schools [24,25]. Comprehensive sexuality education is not systematically integrated into school curricula across China, leaving many young adults to navigate sexual development with minimal formal guidance [26]. More fundamentally, open discussion of sexuality in China remains constrained by deeply rooted cultural taboos, wherein sex continues to be regarded as a private and socially sensitive subject [25]. These constraints are reinforced by the complex interplay of traditional gender norms, evolving social attitudes, and state intervention [27], collectively making many aspects of sexuality, such as premarital sex, difficult to address openly in family and school settings [28].

Consequently, digital platforms do not merely supplement formal education for Chinese college students; they often constitute the primary, and sometimes the sole, source of sexual health information. This reliance persists despite widespread recognition of online information’s limitations. Preliminary evidence suggests that Chinese young adults are acutely aware of the unreliability of online sexual health information yet continue to use it. In this context, the trust paradox is not merely a theoretical curiosity but an empirical reality that demands systematic investigation. What remains unclear is how individuals navigate this paradox in the domain of sexual health, where the consequences of misinformation are severe, trusted alternatives are often unavailable, and the imperative to obtain reliable information coexists with the recognition that available sources are not fully trustworthy.

This qualitative study aims to explore how Chinese college students navigate the trust paradox in digital sexual health information seeking. Specifically, we investigate (1) how participants perceive the reliability of online sexual health information; (2) what factors shape their continued use of online sources; (3) what strategies they develop to manage informational uncertainty; and (4) what costs they incur because of this reliance. By examining these questions, we seek to generate insights that can contribute to a deeper conceptual understanding of how trust is experienced and navigated in digital health information seeking and to inform practical interventions aimed at improving sexual health outcomes among young adults.

## Methods

### Study Design

This study used an interpretive qualitative design using semistructured, in-depth interviews to explore how Chinese college students navigate uncertainty when seeking sexual health information online. An interpretive qualitative approach was considered appropriate because the study aimed not only to document participants’ experiences but also to develop an in-depth understanding of the meanings and practices through which they managed the tension between distrust and continued reliance on digital information [29]. Data were analyzed using Braun and Clarke’s reflexive thematic analysis, an interpretive analytic approach that enables researchers to develop conceptual understandings grounded in patterned meanings across participants’ accounts. The study was reported in accordance with the COREQ (Consolidated Criteria for Reporting Qualitative Research) guidelines [30].

### Participants and Recruitment

Participants were recruited from 8 universities across China using a purposive sampling strategy with maximum variation, complemented by snowball sampling techniques. The eligibility criteria included (1) being an undergraduate student, (2) being between 18 and 25 years of age, (3) having accessed the internet to search for sexual health information at least once, and (4) providing informed consent. We used a maximum variation approach to capture a broad and diverse range of perspectives. Accordingly, we deliberately sought variation across key demographic and contextual dimensions, including gender, geographic region, university type, and academic discipline (health-related majors vs nonhealth majors). This strategy was intended to ensure that our findings reflect the heterogeneity of experiences among Chinese college students, rather than the views of a single subgroup.

Recruitment began through the researchers’ academic networks. Initial participants, who were known to the research team and met the eligibility criteria, were invited to participate. We acknowledge that using personally known contacts for recruitment on a sensitive topic could raise concerns about social desirability bias or reluctance to disclose. To mitigate this, we used several strategies: first, initial participants were informed that the research team’s role was strictly professional and that they could decline participation without any consequences to their academic or personal relationships; second, interviews were conducted by telephone rather than in person, which prior research suggests may actually reduce social pressure and encourage disclosure on sensitive topics [31,32]; third, interviewers explicitly emphasized confidentiality and nonjudgment before asking any sensitive questions; and fourth, we supplemented initial recruitment with snowball sampling through peer referrals, which has been shown to foster trust and reduce perceived judgment when discussing sensitive health topics. Given the sensitive nature of the topic, we supplemented this initial recruitment with snowball sampling: initial participants were asked to share the study information with peers across various universities via word-of-mouth and social media platforms, primarily WeChat. This peer-referral approach was deliberately chosen to foster trust, reduce social desirability bias, and encourage participation among individuals who might otherwise be hesitant to discuss sexual health topics.

The universities represented a range of types, including Central South University, Xinjiang Medical University, Xinjiang University of Science and Technology, Yangzhou University, Nanjing Audit University, East China University of Science and Technology, Xuzhou University of Technology, and Changsha Social Work College. All eligible candidates who expressed interest and provided informed consent were enrolled. Recruitment continued concurrently with data collection until we achieved adequate variation across the target dimensions and data saturation was reached, that is, no new themes or insights emerged from subsequent interviews.

### Data Collection

Data were gathered through semistructured, in-depth interviews held in January 2026. The interview guide was designed based on a thorough literature review and piloted with 2 undergraduate students who were not included in the final sample. The purpose of the pilot was to assess question clarity, flow, and cultural appropriateness, not to achieve saturation. Both pilot participants confirmed that the questions were understandable and relevant; no major changes were required, so the guide was used without modification. The interview topic focused on participants’ experiences with online sexual health information seeking, specifically exploring: (1) how they discovered and assessed information across various digital platforms; (2) their views on information reliability and experiences with conflicting content; (3) strategies they used to evaluate credibility and manage uncertainty; and (4) the tension between distrust and continued reliance on online sources. Interviews also included questions on face-to-face communication about sexual health and the role of cultural factors in shaping information-seeking behaviors.

Interviews were conducted by the first author, a female postdoctoral researcher with extensive experience in qualitative research and sexual health education. All interviews were carried out in Mandarin Chinese, via telephone, and were audio-recorded. The telephone method was chosen to ensure participant comfort and to reduce the impact of visual presence, which could discourage open discussion about sexual health in the Chinese cultural context. This approach helped minimize judgment and social desirability bias. Extensive methodological literature supports this choice: telephone interviews on sensitive topics have been found to encourage disclosure of potentially embarrassing or stigmatized information, as the absence of visual presence can reduce social pressure and allow participants to feel more at ease [31,32]. In fact, some studies suggest that telephone interviews may produce data of comparable depth and quality to face-to-face interviews when discussing sensitive subjects [31,32]. The telephone method also enabled us to reach geographically diverse participants across multiple provinces without imposing travel burdens. We acknowledge, however, that telephone interviews have limitations, including the loss of nonverbal cues and the potential for reduced rapport-building. To mitigate these, the interviewer engaged in active listening, used verbal affirmations, and established rapport before delving into sensitive questions. All interviews were conducted in a private setting with no interruptions, and participants were encouraged to choose a quiet environment for the call.

The duration of the interviews ranged from 30 to 90 minutes. Saturation was monitored throughout the data collection process, with the final interviews showing no emergence of new themes or insights. With participants’ consent, all interviews were recorded, transcribed verbatim in Chinese, and anonymized with pseudonyms. Quotations used in the manuscript were translated from Chinese to English by the first author (YS), a bilingual researcher fluent in both languages, and reviewed by the second author (YZ) to ensure accuracy and preserve the original meaning. Any discrepancies in translation were resolved through discussion between the 2 translators.

### Data Analysis

Data were analyzed using Braun and Clarke’s reflexive thematic analysis [33], an interpretive analytic approach that facilitates the identification and interpretation of patterned meanings across participants’ accounts. Analysis followed six recursive phases: (1) familiarization with the data, (2) generating initial codes, (3) constructing candidate themes, (4) reviewing themes, (5) defining and naming themes, and (6) producing the final report. Throughout the analytic process, movement between the data, coding, theme development, and interpretation was iterative rather than linear, with the aim of developing a richer conceptual understanding of how participants navigated the tension between distrust and continued reliance on digital sexual health information.

The analysis was primarily conducted by the first author, with ongoing discussions among the research team to ensure analytic rigor and reflexivity. Transcripts were read repeatedly to achieve familiarity with the data, and initial coding was conducted inductively and remained flexible throughout the analytic process. Rather than applying a predetermined coding framework, codes and candidate themes were continuously refined through iterative engagement with the entire dataset. Themes were reviewed, compared, and refined to ensure that they captured coherent patterns of shared meaning while remaining closely connected to participants’ accounts. Reflexive practices were used throughout the analysis to critically consider how the researchers’ disciplinary backgrounds, assumptions, and experiences might shape interpretation. A reflexive journal was maintained to document analytic decisions and support ongoing critical reflection.

Member checking was conducted with 5 participants from diverse backgrounds (varying in gender, academic discipline, and geographic region) to explore whether our preliminary interpretations resonated with their experiences and to identify areas requiring clarification. This number was selected pragmatically to ensure representation across key participant characteristics while avoiding unnecessary participant burden and is consistent with recommendations that member checking should be purposive rather than exhaustive [34]. Participants’ feedback informed further refinement of our interpretations but was not treated as verification of a single correct account.

To minimize confirmation bias, several strategies were used throughout the analytic process. Initial coding remained inductive and data-driven, with codes developed from participants’ accounts rather than from preexisting theoretical frameworks. Divergent and negative cases were actively sought and incorporated into theme development to challenge emerging interpretations. Regular analytic discussions among the research team encouraged consideration of alternative interpretations of the data. Together, these strategies strengthened the credibility of our interpretations by helping ensure that themes remained closely connected to participants’ accounts while acknowledging the interpretive role of the researchers.

### Trustworthiness

Several strategies were used to enhance the trustworthiness of the study [34,35]. Credibility was enhanced through prolonged engagement with the data, reflexive discussions within the research team, and participant feedback that informed refinement of our interpretations. Transferability was supported through rich description of the participants, research context, and findings, enabling readers to judge the applicability of the findings to other settings. Dependability was strengthened through systematic documentation of the research process, including interview guides, analytic decisions, reflexive journals, and analytic memos. Confirmability was supported through ongoing reflexive practice, maintaining an audit trail, and ensuring that interpretations remained closely connected to participants’ accounts while acknowledging the interpretive role of the research team.

### Ethical Considerations

This study received approval from the institutional review board of the affiliated institution (approval number KJXY-LLSC 2026-No.10). Participants were informed about the study’s purpose, procedures, confidentiality, and their right to decline any question or withdraw at any time without consequence. Written informed consent was obtained before all interviews. Identifying details were removed from transcripts, and pseudonyms were used in the final report. No financial compensation or reimbursement was provided, as all interviews were conducted by telephone and participation was entirely voluntary. Following the interviews, participants were offered the opportunity to consult the research team about any sexual health concerns that arose during or after the interview. This arrangement was reviewed and approved by the institutional review board as part of the study protocol, which determined that providing evidence-based sexual health information constituted a minimal-risk educational service rather than clinical advice. No adverse events were reported.

## Results

### Basic Characteristics

A total of 22 participants were included in the study. The sample comprised 12 female and 10 male individuals, with the majority identifying as Han ethnicity, alongside a small number of participants from Uyghur and Tujia ethnic backgrounds. Participants were drawn from multiple universities across China, including institutions in Xinjiang as well as other regions such as Jiangsu and Hunan, ensuring diversity in geographical and educational contexts. The sample included students from a range of academic disciplines: 12 participants from health-related majors (eg, nursing, preventive medicine, and public health) and 10 participants from nonhealth fields (eg, finance, marketing, and engineering). Participants were distributed across different academic years, from first-year undergraduates to senior students, with most in their second and third years of study. This diversity in gender, ethnicity, academic background, and institutional affiliation provided a broad perspective on students’ experiences of seeking online sexual health information. Detailed participant characteristics are presented in Table 1.

**Table 1:** Participant characteristics (N=22).

Characteristic			Value, n
Gender			
	Female	12	
	Male	10	
Ethnicity			
	Han	18	
	Uyghur	3	
	Tujia	1	
Academic year			
	First-year	4	
	Second-year	7	
	Third-year	7	
	Fourth-year	4	
Academic discipline			
	Health-related majors (nursing, preventive medicine, or public health)	12	
	Nonhealth majors (finance, marketing, or engineering)	10	
University geographic region^a^			
	Central South China (Hunan)	6	
	East China (Jiangsu)	7	
	Northwest China (Xinjiang)	9	

^a^Geographic regions are reported by university location.

### Overview of Themes

#### Overview

This study explored how Chinese college students navigate the tension between distrust and reliance when using online sexual health information. Analysis of 22 interviews generated four interconnected themes: (1) perceiving a fragmented and unreliable information landscape; (2) the paradox of incomplete trust yet continued reliance; (3) strategies for navigating information uncertainty; and (4) the hidden costs of navigation. An overview of these themes is presented in Textbox 1.

**Overview of themes.**

**Theme 1: fragmented and unreliable information landscape**

- Participants encountered abundant but often contradictory, superficial, and difficult-to-apply information.

**Theme 2: incomplete trust yet continued reliance**

- Participants relied on online information despite doubts, due to risk concerns and lack of alternatives.

**Theme 3: strategies for navigating information uncertainty**

- Participants used adaptive practices to evaluate and use uncertain information in practical decision-making.

**Theme 4: hidden costs of navigation**

- Information seeking often resulted in confusion, fatigue, and abandonment.

#### Theme 1: A Fragmented and Unreliable Information Landscape

##### Overview

Participants consistently described the digital sexual health information environment as abundant yet difficult to trust. Although information was abundant and easy to obtain, participants frequently encountered superficial content, contradictory recommendations, and information that lacked practical relevance to their personal situations. Rather than facilitating informed decision-making, these challenges often generated uncertainty and prompted participants to engage in ongoing evaluation of information quality and credibility.

##### Abundance Without Depth

Participants generally agreed that sexual health information had become increasingly available online. However, greater availability did not necessarily translate into greater usefulness. Many felt that online content provided broad explanations of health topics but offered limited guidance for addressing specific concerns or making decisions in real-life situations. As one participant explained:

There is a lot of information online, but most of it is very shallow. It tells you what something is, but it doesn’t tell you what to do if it actually happens to you.

P16

Several participants distinguished between general educational content and information that could be applied to their own circumstances. While introductory explanations were easy to find, situation-specific guidance was often perceived as lacking.

It’s easy to find general explanations, but when it comes to your own situation, there’s nothing very specific.

P14

Participants also questioned the quality of information available online. Some perceived substantial variation in content quality and expressed concerns about the motivations of information providers.

The quantity is growing, but the quality is mixed. Some people genuinely want to educate, while others just want to gain followers or even mislead.

P11

Overall, participants described an information environment in which access was rarely a problem, but identifying information that was trustworthy, detailed, and practically useful remained challenging.

##### Contradictory Information and Uncertainty

A common experience across interviews was encountering contradictory information from different online sources. Participants reported receiving conflicting explanations, recommendations, and interpretations regarding the same sexual health issue.

Different sources say completely different things. One says yes, another says no.

P7

Contradictions were particularly difficult to manage when they originated from sources that participants perceived as similarly credible. For instance, disagreements among different doctors, a source type many participants viewed as professionally authoritative, were especially challenging to navigate. As one participant noted:

Different doctors give different answers. One says this, another says that. I have no idea which one is correct.

P21

In such situations, participants often struggled to determine which information should be trusted. For some, exposure to conflicting information increased uncertainty rather than resolving it.

The more I searched, the more confused I became. I eventually gave up.

P13

When I see different opinions on the same issue, I don’t know which one is true, so I just stop.

P2

However, participants did not respond to contradiction in the same way. While some disengaged from further searching, others adopted more active verification strategies by consulting additional websites, platforms, or experts.

When I see different opinions, I don’t stop. I just check more places.

P3

These findings suggest that contradictory information often triggered additional information seeking rather than immediate resolution. Nevertheless, even extensive verification efforts did not always provide participants with a clear answer.

##### High Matching But Low Resolution

Participants frequently described a gap between finding information related to their questions and obtaining information that actually resolved their concerns. Search results were often perceived as relevant to the topic being searched, yet insufficient for guiding action or decision-making. One participant summarized this discrepancy as follows:

Maybe 80 to 90 percent of what I find matches what I’m looking for, but only 30 to 40 percent actually solves my confusion.

P21

Another participant expressed an even more critical view:

Overall, less than 20 percent is truly useful.

P19

Across interviews, participants consistently distinguished between relevance and usefulness. Information was considered useful not only when it was accurate, but when it provided actionable guidance that helped them determine what to do next. Content that merely described a condition or presented general information was often viewed as insufficient for addressing personal concerns.

When asked to estimate more broadly, most participants reported that approximately 70%-80% of search results matched their initial query, whereas only 30%-40% helped them make decisions or resolve uncertainty. This discrepancy highlights a recurring challenge within participants’ information-seeking experiences: although relevant information was readily available, actionable answers were much harder to obtain.

#### Theme 2: Incomplete Trust Yet Continued Reliance

##### Overview

Despite expressing concerns about the credibility of online sexual health information, most participants continued to rely on it. This reliance was not driven by confidence in the accuracy of the information but by the practical need to obtain guidance in situations where uncertainty, urgency, or limited alternatives left few other options. Participants therefore developed ways of using online information while acknowledging its limitations.

##### Risk-Oriented Decision-Making

Participants often described making decisions based on perceived risk rather than certainty. When evaluating online sexual health information, many focused less on whether the information was completely accurate and more on the potential consequences of ignoring it. As one participant explained:

Rather than ignore it, I’d rather believe it. Having an extra precaution is better than none.

P9

Similarly, another participant noted:

Even if it might not be true, taking extra precautions is better than not taking any at all.

P4

These accounts suggest that participants frequently adopted a precautionary approach when faced with uncertainty. In sexual health contexts, where concerns often involved potential infections, unintended pregnancy, or delayed treatment, the perceived consequences of inaction were viewed as more serious than the risks associated with acting on information that might later prove inaccurate. As a result, participants were often willing to follow preventive advice despite reservations about its reliability.

Several participants described taking precautionary measures after encountering online information about symptoms or potential health risks. Even when they were uncertain about the accuracy of the information, they felt that taking preventive action was a safer option than disregarding the advice altogether. Continued reliance on online information therefore reflected not complete trust, but a pragmatic response to perceived vulnerability and uncertainty.

##### Lack of Offline Alternatives

Participants also emphasized that their reliance on online information was shaped by the limited availability of alternative sources of sexual health guidance. For many, discussing sexual health with family members was difficult or discouraged, leaving the internet as one of the few accessible sources of information.

I don’t discuss these things with my family...so I still rely on online information. It’s all I have.

P18

Family silence surrounding sexual health was a recurring experience across interviews. Participants described sexual topics as uncomfortable, sensitive, or inappropriate to discuss within the family setting. In some cases, they anticipated negative reactions if such conversations were initiated.

In traditional families, it’s hard to bring up these topics, and sometimes you might even be scolded. So, the internet becomes indispensable.

P11

Beyond family relationships, participants also reported limited willingness to seek information from friends, peers, or other offline contacts. Concerns about embarrassment, judgment, or misinformation often discouraged such discussions.

Because there’s no one around me to talk to, my reliance on the internet has become higher and higher.

P7

You can’t discuss it with others most of the time, so you have no choice but to search and find information on your own.

P15

Across interviews, participants repeatedly described online information seeking as a necessity rather than a preference. Their continued reliance on digital sources reflected not only the accessibility of online information but also the absence of trusted and comfortable offline alternatives.

##### The Internet as a Reference Rather Than an Authority

Although participants relied heavily on online information, they rarely viewed the internet as a fully authoritative source. Instead, many described using online information as an initial reference point that required further evaluation and verification. One participant compared the internet to a reference book:

To me, the internet is like a reference book. I gain knowledge from it, but mostly for reference. Its professionalism still needs verification.

P18

This view was echoed by another participant:

It tells me that information exists, but I still need to check it myself.

P2

Rather than accepting information at face value, participants often treated online content as a reference point requiring subsequent judgment, rather than a definitive answer. This allowed them to continue using online resources while maintaining skepticism. They described comparing information across multiple websites, consulting additional sources, and reserving judgment when information appeared inconsistent. This approach allowed them to continue using online resources while maintaining a degree of skepticism about their accuracy.

Importantly, participants did not see reliance and distrust as mutually exclusive. Instead, they navigated online information by balancing the practical benefits of accessibility with ongoing efforts to assess credibility. The internet was valued as a useful source of information, but final judgments about what to believe or how to act were typically made only after further consideration.

Overall, these findings indicate that continued reliance on online sexual health information persisted despite concerns about trustworthiness. Participants managed this tension through precautionary decision-making, reliance born of limited alternatives, and the use of the internet as a reference rather than an unquestioned authority. These strategies enabled continued engagement with digital information while accommodating persistent uncertainty about its reliability.

#### Theme 3: Strategies for Navigating Information Uncertainty

##### Overview

Given the uncertainty and limited trust described in the previous themes, participants developed a range of strategies to evaluate and use online sexual health information. These strategies did not eliminate uncertainty but helped participants make information sufficiently actionable for practical decision-making. Importantly, these strategies were not isolated tactics but formed an interconnected repertoire of practices that collectively reflected how participants attempted to make uncertain information sufficiently usable for practical decisions. Four interconnected strategies were commonly reported: cross-platform verification, credibility assessment, the use of experiential knowledge, and platform differentiation.

##### Cross-Platform Verification

The most frequently described strategy was cross-platform verification. Rather than relying on a single source, participants routinely compared information across multiple platforms and treated consistency across sources as an indicator of reliability.

I compare all the search results. If the majority say the same thing, I assume it’s probably correct.

P13

Participants often described this process as a normal part of information seeking rather than a deliberate verification effort. Information from one source was rarely accepted without checking additional sources.

I never rely on just one source. I always check several.

P3

Many participants followed relatively structured verification routines that involved consulting different types of platforms for different purposes.

I ask the AI, then check browsers, then look at Xiaohongshu to see others’ experiences.

P15

Rather than seeking a single authoritative answer, participants combined information from multiple sources to build a more complete understanding of a topic. Verification therefore functioned as an ongoing process of comparison and triangulation rather than confirmation from any single source.

##### Assessing Credibility Beyond Credentials

Participants also evaluated the credibility of individual sources. Professional credentials, such as medical qualifications or physician-affiliated accounts, were generally viewed as positive indicators of trustworthiness, but credentials alone were rarely considered sufficient.

If it’s from a doctor’s account, I feel it’s more reliable. But I still make my own judgment.

P1

Many participants reported paying attention to the presentation and style of information. Content perceived as balanced, informative, and educational was viewed more favorably than content that appeared sensationalized or primarily designed to attract attention.

I look at whether it’s clickbait or genuinely informative.

P11

At the same time, participants remained cautious even when information came from seemingly authoritative sources.

Nowadays, a lot of information online is fake, even from so-called professionals.

P3

Concerns about commercial influence were also common.

I’m worried about being led astray by marketing accounts. I stay cautious.

P11

These findings suggest that participants relied on multiple credibility cues simultaneously. Professional expertise was valued, but judgments about trustworthiness also incorporated perceptions of intent, presentation style, and consistency with other information sources.

##### Experiential Knowledge From Comment Sections

Beyond formal information sources, many participants actively consulted comment sections to access the experiences of other users. Comment sections were viewed as providing practical insights that complemented more formal educational content.

When people share their own experiences in the comments, it feels easier to understand. It feels more real than just listening to an expert talk.

P1

Participants often valued peer experiences because they provided concrete examples of how others had interpreted symptoms, sought treatment, or managed health concerns. Such experiences helped participants relate information to their own circumstances.

Some participants also viewed repeated reports from multiple users as a form of informal validation.

If many people in the comments say they tried it and it worked, I feel more confident. It’s not just theory, as it’s what happened to people like me.

P12

However, participants were also aware of the limitations of user-generated content.

People in the comments just make things up casually. I don’t take it too seriously.

P4

Sometimes comments are helpful, but sometimes they’re just random opinions.

P11

As a result, comment sections were rarely treated as authoritative sources. Instead, they were used as supplementary resources that helped participants contextualize and interpret information obtained elsewhere.

#### Platform Differentiation

Participants demonstrated a clear awareness that different digital platforms served different informational functions. Rather than viewing online sources as interchangeable, they selected platforms according to the type of information they were seeking. Video-based platforms such as Douyin and Kuaishou were often used to obtain visual explanations and simplified descriptions of health topics. Participants reported that video content was easier to understand, particularly for topics involving anatomy, symptoms, or medical procedures. Social media platforms such as Xiaohongshu were primarily valued for peer experiences and personal narratives. Participants used these platforms to understand how others had experienced similar situations and to identify practical suggestions that were not always available in formal health information. AI-powered tools such as DeepSeek and Doubao were appreciated for their speed, convenience, and ability to answer follow-up questions. Participants also noted that AI allowed them to ask sensitive sexual health questions without fear of embarrassment or judgment. However, AI-generated information was rarely accepted without further verification. Search engines and physician-oriented websites were generally perceived as sources of more professional or authoritative information. Participants often used these sources to verify information encountered on social media or through AI tools.

Table 2 summarizes the major platform categories identified by participants and their perceived functions. These findings indicate that participants developed sophisticated strategies for navigating online sexual health information. Rather than relying on any single source, they actively combined information across platforms, evaluated credibility using multiple cues, incorporated experiential knowledge, and matched different platforms to different informational needs. This differentiation of platforms into functional categories is itself a form of expertise. These practices enabled participants to continue using online information despite persistent uncertainty about its reliability.

**Table 2:** Platform categories, perceived functions, and illustrative participant descriptions.^a^

Platform category	Examples	Perceived function	Illustrative participant descriptions
Video-sharing platforms	Douyin and Kuaishou	Visual, intuitive explanations; engaging and accessible formats that are easier to understand than text-heavy content	“Douyin has videos, so they’re more vivid and easier to understand.” (P13)
Text-based social platforms	Xiaohongshu (Red Note)	Real, peer-shared experiences; efficient information scanning via titles and tags; perceived authenticity of user-generated content	“Xiaohongshu has real experiences shared by users, and the comments feel more genuine.” (P17)
AI-powered chatbots	Doubao and DeepSeek	Quick, direct answers; ability to ask follow-up questions interactively; perceived as neutral and nonjudgmental	“I ask the AI, then check browsers, then look at Xiaohongshu to see others’ experiences.” (P15)
Search engines/browsers	Baidu and other search engines	Professionally sourced answers (eg, from doctors); perceived as more authoritative than social media	“On Baidu, you get answers directly from doctors, which feels more professional.” (P3)
Podcasts	—^b^	In-depth, long-form content; often humorous and engaging; suitable for background listening	“Podcasts are great, as they go into depth and are actually entertaining to listen to.” (P8)

^a^Platform categories reflect thematic patterns derived from participants’ descriptions of their information-seeking practices. The table is intended to illustrate the range of perceived platform affordances, not to quantify usage frequency across the sample. The podcast category (mentioned by 1 participant) is reported for completeness but should be considered illustrative rather than representative of common practice.

^b^Not available.

#### Selective Silence

Although participants actively searched for and consumed sexual health information online, few reported contributing content or participating in public discussions. Most described themselves as information consumers rather than information contributors.

I don’t think I’m qualified. I’m afraid of saying something wrong and misleading others.

P4

Many participants felt they lacked sufficient expertise to provide advice or share information publicly. Concerns about spreading inaccurate information discouraged them from posting, commenting, or answering others’ questions. Others were more concerned about the potential social consequences of participation.

I worry about being criticized or countered. Sometimes people get attacked online for sharing their experiences.

P15

Participants described online discussions as potentially confrontational, particularly when discussing sensitive topics such as sexual health. Fear of criticism, disagreement, or negative reactions often reduced their willingness to engage publicly.

Several participants explicitly characterized themselves as observers rather than contributors.

I just read and never participate. I’ve always stayed on the sidelines.

P12

I just watch and never say anything. If I want to discuss, I’d rather talk with friends in person.

P14

These accounts suggest that participants preferred to obtain information from online communities while limiting their own visibility within them. Although they valued the experiences and perspectives shared by others, they generally chose to engage through observation rather than direct participation. Discussions about sexual health were often perceived as more comfortable in private and trusted interpersonal settings than in public online spaces.

Overall, participants’ information practices were characterized by active information consumption but limited content contribution. This pattern highlights an important distinction between accessing digital health information and participating in digital health communities. While online platforms provided valuable opportunities for learning and information seeking, most participants remained observers rather than active contributors.

#### Theme 4: The Hidden Costs of Navigation

##### Overview

Although participants developed a range of strategies to navigate online sexual health information, these strategies often required substantial effort. Many described information seeking as time-consuming, mentally demanding, and sometimes emotionally frustrating. Three interconnected challenges emerged across interviews: information overload, decisional paralysis, and search abandonment.

##### Information Overload

A common experience among participants was feeling overwhelmed by the volume of information encountered during searching. While the availability of information was initially viewed as beneficial, participants often found it difficult to manage the large number of sources, platforms, and search results they encountered.

I was searching for one thing, but as I scrolled, I found something else interesting. I opened several pages and eventually forgot what I was originally looking for.

P14

Participants frequently described being drawn into multiple search pathways, making it difficult to maintain focus on their original question.

You start with one question, and suddenly you have ten tabs open and you’re not even sure which one was the original.

P18

The process of comparing information across platforms, a strategy many participants used to verify information, often increased the amount of content that needed to be reviewed and interpreted. As a result, information seeking frequently became more time-consuming and cognitively demanding than participants had anticipated.

Many participants reported feelings of frustration and fatigue after extended searching. Rather than creating clarity, the abundance of information sometimes made it more difficult to identify useful answers and determine what information deserved attention.

##### Decisional Paralysis

Beyond the challenge of information volume, participants also struggled with conflicting information. Contradictory recommendations from different sources often made it difficult to decide which information should guide action.

Different sources say completely different things. One says yes, another says no. How am I supposed to know which one to follow?

P7

Participants frequently described situations in which multiple sources appeared equally credible yet offered opposing advice. In these circumstances, additional searching often increased uncertainty rather than resolving it.

I found all sorts of explanations. The more I searched, the more confused I became. I eventually gave up without deciding what to believe.

P13

Several participants reported feeling unable to make decisions because they lacked confidence in their ability to judge between competing claims. Although they had access to a large amount of information, they often felt unable to determine which information was most accurate or applicable to their situation.

Information overload and contradictory information frequently interacted. Participants described having access to more information than ever before while simultaneously feeling less certain about what action to take. As a result, information seeking did not always achieve its intended purpose of reducing uncertainty and supporting decision-making.

##### Search Abandonment

For some participants, prolonged searching eventually led to search abandonment. When information remained confusing, contradictory, or difficult to evaluate, participants sometimes chose to stop searching before their questions had been fully resolved.

If it doesn’t work, I stop there. I don’t keep pushing.

P2

Others described weighing the effort required for continued searching against the likelihood of obtaining useful information.

If I can’t find what I need, I give up. It’s not worth the time and frustration.

P5

Search abandonment was particularly common when participants encountered persistent contradictions across sources.

If one says yes and another says no, I can’t just take the middle value. So, I just stop looking.

P22

For these participants, further searching was often perceived as unlikely to provide a definitive answer and more likely to generate additional confusion.

However, participants varied in their willingness to persist. Some abandoned searches relatively quickly, whereas others continued exploring alternative keywords, platforms, or information sources when they considered the topic important.

If I can’t find it after a few tries, I give up. But if it’s really important, I’ll try different keywords or platforms.

P1

I keep searching until I find what I need. I don’t stop easily.

P3

Despite these differences, even highly persistent participants acknowledged that extended searching did not always lead to satisfactory resolution. In some cases, prolonged efforts ended with the same uncertainty that had prompted the search in the first place.

Collectively, these findings highlight the hidden costs associated with navigating online sexual health information. Participants invested substantial time and effort in searching, comparing, and evaluating information, yet these efforts did not always produce clarity or confidence. Information overload, contradictory information, and unresolved uncertainty frequently transformed information seeking from a source of empowerment into a source of frustration, illustrating the limits of individual coping strategies within complex digital information environments. The tension between enablement and burden lay at the heart of participants’ experiences: they were simultaneously empowered by their strategies and constrained by the effort those strategies demanded.

In summary, these 4 themes are not arranged as a simple linear progression but rather as a recursive and dynamically interrelated process: structural constraints (Theme 1) and the knowledge-action gap create conditions that necessitate reliance (Theme 2), which in turn drives the active construction of functional trust through adaptive strategies (Theme 3). The cognitive and emotional costs incurred during this process (Theme 4) may, however, feed back into future information-seeking behaviors, potentially reshaping subsequent reliance and strategy selection. This recursive dynamic underscores the nonlinear, iterative nature of navigating digital health information—a complexity that aligns with our earlier critique of linear models of information behavior and highlights the need for more ecologically grounded frameworks.

## Discussion

### Principal Findings

This study examined how Chinese college students navigate online sexual health information in the face of persistent uncertainty. Participants described an information environment characterized by abundant but often contradictory and difficult-to-apply information. Despite expressing concerns about the credibility of online sexual health content, they continued to rely on digital sources because of limited offline alternatives and the perceived risks associated with inaction. To manage uncertainty, participants developed a range of strategies, including cross-platform verification, credibility assessment, consultation of peer experiences, and platform-specific information seeking. However, these strategies often required considerable effort and were associated with information overload, decisional paralysis, and search abandonment.

These findings reveal a dynamic process through which individuals navigate the tension between distrust and reliance. Rather than making a simple distinction between trusted and untrusted information, participants actively worked to make information usable under conditions of uncertainty. Based on these findings, we conceptualize functional trust as an interpretive conceptualization of how individuals make uncertain information sufficiently actionable to support decision-making despite ongoing uncertainty about its accuracy. Rather than representing a stable belief in source credibility, functional trust explains how participants rendered uncertain information usable through active engagement with a fragmented and often unreliable digital information environment.

Before proceeding, it is important to clarify how functional trust differs from related constructs in information science and health communication. Wilson’s concept of “satisficing” describes a cognitive economy strategy in which users settle for information that is “good enough” given time and resource constraints [36]. While functional trust also involves pragmatic trade-offs, it goes beyond satisficing by emphasizing the active construction of action-oriented confidence, which means users do not merely accept sufficient information; they actively verify, compare, and validate across sources to render uncertain information usable. Unlike passive coping frameworks that emphasize accommodation to structural constraints (eg, Chatman’s life-world perspective [37]), functional trust is more agentic: participants in our study did not simply accept the limitations of their environment but developed deliberate, cross-platform strategies to evaluate and act on information. Finally, notions of “provisional” or “instrumental” trust in information science typically refer to temporarily accepting a source’s credibility for a specific purpose. Functional trust, however, is not a judgment about the source but an interpretive orientation through which users make information sufficiently actionable, even when source credibility remains uncertain. This distinction shifts the analytical focus from source properties to user practices, making functional trust a complementary rather than redundant concept.

A key contribution of this study is the identification of a gap between informational relevance and practical usability in digital sexual health contexts. While participants reported relatively high rates of information matching, their ability to translate this information into actionable decisions remained limited. We refer to this disconnect as the knowledge-action gap—that is, the discrepancy between finding information that appears relevant to one’s query and being able to convert that information into concrete, actionable decisions or behaviors. This knowledge-action gap suggests that existing digital resources are more effective in addressing “what is” questions than “how to” concerns that require context-sensitive, situational guidance. Importantly, this gap helps explain why participants continued to engage in repeated searching and cross-platform verification despite extensive exposure to information. The challenge, therefore, lies not only in access but in the translation of knowledge into action.

Within this context, functional trust helps explain how participants were able to act under conditions of persistent uncertainty. Rather than relying on information because they believed it to be objectively accurate, participants made information sufficiently usable through a series of adaptive practices. These findings challenge linear models of information behavior that position trust as a prerequisite for use [36,38]. Instead, our findings suggest a more iterative and pragmatic pathway in which use precedes, and in some cases substitutes for, belief. In this sense, functional trust reflects this pragmatic orientation. Participants used a set of interrelated practices that enabled them to make uncertain information sufficiently actionable despite ongoing uncertainty. We interpret these practices collectively as reflecting what we term functional trust. Cross-platform verification, credibility assessment, experiential validation, and platform differentiation constitute the empirical basis for this conceptualization rather than evidence of an underlying psychological construct.

Participants engaged in cross-platform verification, treating convergence across independent sources as a proxy for reliability. They evaluated not only formal credentials but also communicative tone and perceived intent, while drawing on experiential knowledge, particularly from comment sections, to contextualize and appraise expert advice. Many participants sought confirmation of expert information through the experiences of others with similar concerns, reflecting a shift in which knowledge was not simply accepted from authoritative sources but interpreted through peer experience. Participants also developed a nuanced understanding of platform-specific affordances, recognizing that different platforms were better suited to different types of information and informational needs.

Our concept of functional trust should be distinguished from related concepts such as information efficacy and health behavior strategies. Information efficacy refers to confidence in one’s ability to locate, evaluate, and use health information, whereas functional trust concerns how individuals come to regard uncertain information as sufficiently actionable despite persistent uncertainty about its accuracy. Likewise, health behavior strategies, such as seeking second opinions or delaying decisions, are specific adaptive practices. By contrast, functional trust is an interpretive conceptualization that explains how these practices collectively enable individuals to navigate uncertainty while continuing to rely on digital sexual health information. Viewed collectively, these findings illustrate a pragmatic, experience-driven approach to making health information usable in contexts where certainty is unattainable. Figure 1 presents an interpretive conceptual model summarizing how Chinese college students navigated the digital sexual health information environment and how the concept of functional trust emerged from these adaptive practices within broader structural constraints.

The model presents an interpretive conceptualization of how Chinese college students navigated uncertainty in digital sexual health information seeking. Structural constraints (no offline alternatives, culturally enforced taboos, and precautionary risk logic) contributed to the knowledge-action gap and shaped participants’ reliance on uncertain digital information. Functional trust is conceptualized from 4 interrelated adaptive practices—cross-platform verification, credibility assessment, experiential validation, and platform differentiation—which together enabled participants to make uncertain information sufficiently actionable despite ongoing uncertainty. The model also highlights the cognitive and emotional costs associated with these practices, including information overload and search abandonment.

At the same time, the construction of functional trust was associated with significant cognitive and emotional costs. Many participants reported increased confusion, information overload, and, in some cases, search abandonment. These patterns are consistent with previous evidence linking information overload to search discontinuation [39,40]. These findings complicate the assumption that greater access to information is inherently empowering. Drawing on cognitive load theory [41], these patterns can be understood as the result of excessive processing demands: participants were required to evaluate multiple, often contradictory sources while simultaneously attempting to make decisions. Importantly, these costs may have cumulative effects. Repeated experiences of confusion or unsuccessful searches may reduce information-seeking self-efficacy [42,43], potentially leading to disengagement from future information needs. This highlights the importance of considering not only the accuracy of information but also the cognitive burden associated with navigating complex information environments.

We argue that structural constraints, particularly the absence of accessible offline sexual health communication and culturally reinforced taboos, are not merely background conditions that contextualize information seeking; rather, they are constitutive of functional trust itself. That is, functional trust emerges because of constrained options, not despite them. In an environment where trusted offline alternatives are largely unavailable, the very need to act on uncertain digital information forces users to develop practices that render that information usable. It is plausible that without such constraints, the pragmatic urgency that drives cross-platform verification, experiential validation, and precautionary decision-making might be substantially diminished. The persistence of reliance on online information despite widespread distrust reflects a set of underlying structural conditions, revealing a central paradox: participants continued to act on information they did not fully trust because they had no viable alternatives. Many described a lack of accessible offline sources, including limited communication with family, teachers, or health care providers on sexual health topics. This pattern resonates with Chatman’s [37] concept of an impoverished lifeworld, which depicts a passive accommodation to structural constraints, often resulting in reliance on insiders or small-world norms. In our study, individuals lacked access to such trusted insider sources and had to rely on limited or uncertain information channels, a condition that has been shown to persist in contemporary digital health contexts [44-46]. In this context, digital sources functioned not as preferred options but as necessary substitutes. Decision-making was further shaped by a precautionary logic, in which acting on uncertain information was perceived as safer than inaction when potential risks were involved. This pattern is not simply a matter of poor judgment but an adaptive response to constrained conditions. However, it also creates vulnerability, as precaution-driven use may amplify the influence of inaccurate information when users lack reliable ways to distinguish between valid and invalid claims.

**Figure 1:**
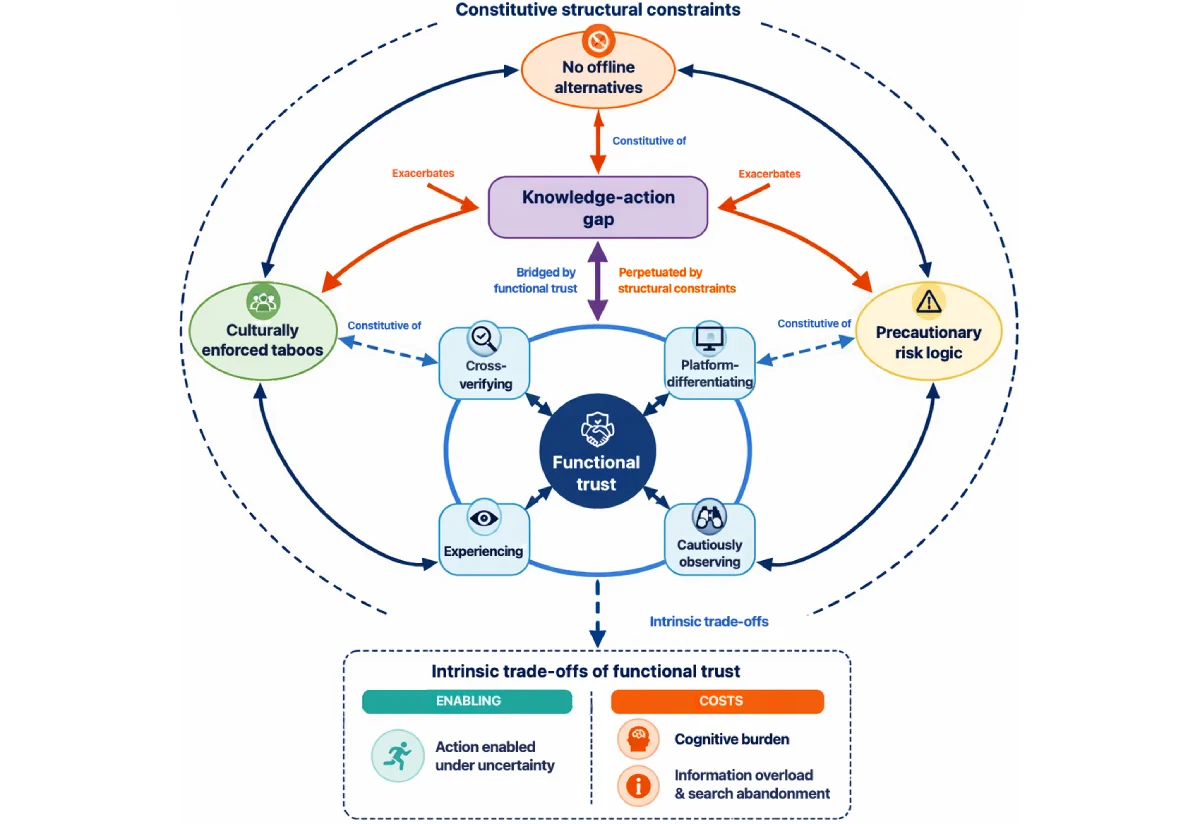
Interpretive conceptual model of how Chinese college students navigated uncertainty in digital sexual health information seeking through functional trust.

These findings have important implications for how digital health literacy is conceptualized. Existing frameworks have tended to emphasize individual skills, such as the ability to evaluate credibility or identify misinformation, often operationalized through measures such as the eHealth Literacy Scale [47,48]. While such skills are clearly important, our findings suggest that they are insufficient in contexts where the information environment itself is structurally unreliable. Participants in this study were not deficient in their critical evaluation of information; rather, they were navigating a system in which even careful evaluation could not guarantee usable outcomes. This calls for a shift from a purely skills-based model of literacy toward a more ecological perspective that recognizes the interaction between users’ evaluative practices and the structural characteristics of the information environment. Within this perspective, functional trust provides an interpretive conceptualization of how individuals continue to make health information sufficiently actionable despite persistent uncertainty.

From a practical perspective, the findings suggest several directions for intervention. First, digital health resources should prioritize actionable guidance alongside general information, addressing not only “what is” but also “what to do.” Second, platforms could support users’ existing verification practices by making consensus more visible, for example, through aggregated summaries or clearly indicated areas of agreement across sources. Third, integrating experiential knowledge with expert information may enhance relevance and usability, provided that such content is appropriately curated. Finally, reducing the cognitive burden of navigation, through clearer organization, structured summaries, and decision aids, may help mitigate the negative consequences identified in this study.

Several limitations should be noted. First, regarding composition and representativeness, the sample was recruited from 8 universities across China using purposive and snowball sampling. Although efforts were made to include institutions of different types and regions, the sample was not randomly selected and may not be fully representative of the broader student population in China. The sample included 12 health-related majors and 10 nonhealth majors. Although this distribution is roughly balanced, health-related students may have greater health literacy, which could influence their information evaluation strategies. We note that an exploratory comparison between these subgroups did not reveal substantial differences in the reported strategies. However, given the small subgroup sizes and the fact that the study was not designed for systematic subgroup comparison, this observation should be interpreted with caution. Future research with larger, balanced samples is needed to examine whether disciplinary background moderates information evaluation strategies and the construction of functional trust.

Second, regarding methodological design, the study relied on self-reported experiences rather than observed behavior, and the cross-sectional design does not capture how information practices evolve over time. Future research should use longitudinal designs and incorporate behavioral data to track information-seeking trajectories. Third, regarding geographic coverage, although participants were recruited from 8 universities spanning Xinjiang, Jiangsu, Hunan, and other provinces, the sample does not include institutions from all regions of China (eg, northeastern and southwestern). Therefore, the findings may not be fully transferable to college students in underrepresented regions. Future research should purposively sample from a wider range of geographic and cultural contexts to enhance transferability. Fourth, regarding analytical scope, while the analysis focused on individual strategies, broader institutional and policy-level factors were not examined in depth. Multilevel analyses of the digital health information ecosystem would be a valuable direction for future research.

Fifth, regarding the educational context of the sample, all participants were university students, a population that may not be representative of young adults with lower educational attainment. The strategies described in this study—weighing competing claims, comparing across multiple sources, and engaging in systematic verification—reflect a degree of critical thinking and information literacy that is more typical of university-educated individuals. Moreover, the snowball sampling method, while useful for accessing a sensitive topic, may have further skewed the sample toward individuals with above-average critical thinking skills or greater confidence in navigating digital information, as participants were more likely to refer peers whom they perceived as articulate or engaged. This potential selection bias may limit the transferability of our findings to broader young adult populations, including those with lower health literacy, less educational exposure, or more limited digital navigation skills. Future research should intentionally recruit participants across a wider range of educational backgrounds to examine how functional trust is constructed among populations with varying levels of critical information literacy. In addition, this study did not systematically examine whether participants distinguished between information originating from Chinese vs non-Chinese sources, nor whether they weighted these sources differently. While a few participants mentioned occasionally consulting English-language materials, the interview guide did not probe this dimension systematically. Given the increasing globalization of digital information flows, future research should investigate how source nationality or platform origin influences credibility perceptions and functional trust construction among Chinese young adults.

Despite these limitations, this study makes 3 key contributions. First, it introduces functional trust as an interpretive conceptualization of how individuals make uncertain digital health information sufficiently actionable despite continuing uncertainty. Second, it identifies a knowledge-action gap that helps explain persistent engagement with digital information despite limited usability. Third, it demonstrates that users’ information practices are shaped not only by individual skills but also by structural constraints, highlighting the need for more integrated approaches to digital health communication.

### Conclusion

In conclusion, this study shows that Chinese college students construct functional trust to act on online sexual health information under conditions of persistent uncertainty. This process is shaped by a gap between informational relevance and practical usability and is fundamentally constituted by structural constraints—namely, the lack of accessible offline alternatives and cultural taboos that leave students with no viable choice but to act on uncertain digital information. While this enables decision-making in the absence of reliable alternatives, it also generates cognitive and emotional costs, including confusion, overload, and disengagement. These findings suggest that improving digital sexual health communication requires not only more accurate information, but also greater attention to how information can be made actionable and cognitively manageable within the constraints users face.

## Abbreviations

COREQ: Consolidated Criteria for Reporting Qualitative Research

## Data Availability

The data that support the findings of this study are available from the corresponding author upon reasonable request.
